# Impact of the Coronavirus Disease 2019 Pandemic on the Ophthalmology Department

**DOI:** 10.3390/jcm11061722

**Published:** 2022-03-20

**Authors:** Ha-Eun Sim, Kyeong-Do Jeong, Je-Hyung Hwang

**Affiliations:** 1 Department of Ophthalmology, Sanggye Paik Hospital, Inje University College of Medicine, Seoul 01757, Korea; s4451@paik.ac.kr; 2 Eyelove Eye Clinic, Seoul 01757, Korea; revolte71@gmail.com

**Keywords:** COVID-19, no-show, ophthalmology

## Abstract

We aimed to evaluate the effects of the coronavirus disease (COVID-19) pandemic on the Ophthalmology Department. This study was based on data collected between January 2019 and November 2021. We divided patients scheduled for eye care during pre-COVID-19 (January–December 2019), early COVID-19 (January–December 2020), and late COVID-19 (January–November 2021) periods. Changes in the outpatient cancellation rate in each department were analyzed and compared in the pre-, early, and late periods. The basic information of cancellation and reason for not visiting the clinic were also analyzed. Overall, 121,042 patients were scheduled to visit the Sanggye Paik Hospital Ophthalmology Department. The overall cancellation rate was 19.13% during pre-COVID-19, 24.13% during early COVID-19, and 17.34% during late COVID-19. The reasons for not visiting the clinic included hospital, patient, and contact factors; hospitalization in other departments and hospitals; and death. The Strabismus/Pediatric Ophthalmology Department showed the highest cancellation rate of 24.21% over three years. There were no significant differences in the causes of hospital visits by period. The COVID-19 pandemic has caused an overall decrease in the number of ophthalmic outpatients. However, after about a year, the number of outpatients in these departments recovered to the level before the COVID-19 outbreak.

## 1. Introduction

Severe acute respiratory syndrome coronavirus 2 is a highly infectious pathogen that causes coronavirus disease (COVID-19), which was first reported in Wuhan, China on December 2019 [1]. COVID-19 has caused serious social and economic losses internationally [2]. Nearly two years have passed since the first COVID-19 case was confirmed on 20 January 2020, in South Korea [3]. To reduce the spread of COVID-19, and to protect hospital facilities, numerous countries, including South Korea, implemented restrictions on population movement, fight restrictions, and social distancing, and patients likewise cancelled or deferred clinical care [4,5].

According to a World Health Organization (WHO) survey, more than half of the countries have partially or completely disrupted services for non-communicable diseases. Although the WHO recommends minimizing facility-based care to reduce the risk of COVID-19, the most common causes for disrupted services were cancellations of planned treatments [6].

Ophthalmology has been reported as one of the areas most affected by COVID-19 [4]. Previous studies reported that 72.5% of ophthalmic institutes had completely stopped all clinical work during the nationwide lockdown period [7]. The no-show rates at outpatient ophthalmological services also increased to 33–40.7% during the pandemic period [4,8].

This retrospective cohort observational study aimed to assess the impact of COVID-19 on ophthalmic outpatients. We investigated the no-show rate of pre-COVID-19, early COVID-19, and late COVID-19 periods, and focused on patient basic and disease characteristics associated with no-show.

## 2. Materials and Methods

### 2.1. Study Design and Ethics

This retrospective cohort observational study was conducted at the Department of Ophthalmology at the Sanggye Paik Hospital of the Inje University in Seoul, Korea. This study was approved by the Institutional Review Board of the Sanggye Paik Hospital (approval number: SGPAIK 202201009). This research adhered to the tenets set forth in the Declaration of Helsinki.

### 2.2. Subjects

This study included patients who visited the ophthalmology outpatient clinic at the Sanggye Paik Hospital from January 2019 to November 2021. The first COVID-19-confirmed case in Korea occurred in January 2020. Therefore, the patients were divided by their appointment date into three periods: pre-COVID-19 period from January 2019 to December 2019, COVID-19 period from January 2020 to December 2020, and late COVID-19 period from January to November 2021. In addition, the number and cancellation rate of outpatients were analyzed by dividing them according to the Departments of Retina, Strabismus/Pediatric Ophthalmology, Cornea, Glaucoma, and General Ophthalmology. Outpatient cancellation included voluntary cancellation of the patient, no-show on the appointment date without notifications, and visiting the clinic after changing the original appointment to more than a month after.

### 2.3. Outcome Analysis and Variables

The number of outpatient appointments and no-show rates were compared according to the periods, departments, and ages. In addition, the default rate was compared by gender, residence, and age group (under 20/40/60/80). The patient’s detailed address could not be collected to protect the patient’s personal information. Therefore, the patient’s residence was classified into cases in Nowon-gu (area 35.44 km^2^), which is the same administrative district as the hospital, and in other areas. The reasons for not visiting were also compared by period. The reasons for not visiting the clinic included hospital factors, such as no improvement in subjective symptoms, visiting another ophthalmic clinic, complaints about medical staff (lack of explanation, unfriendliness), dissatisfaction with administrative procedures (medical record copy, medical certificate, inconvenience in receiving), complaints on facilities of the hospital (elevators, parking), delays in examination and treatment, demands for expert ophthalmic clinics, and delays in medical image interpretations. In addition, there were patient factors, such as personal reasons (family events, business trips, overseas trips), spare medications, lack of proper appointment time, improvement of symptoms, simple cancellations, unwanted examinations, moving to other regions, cost burden, and forgetting the appointment. In addition, there were errors in contact numbers, failure to receive phone calls, deaths, hospitalization in other departments, and hospitalization in other hospitals. In addition, the effect of the nationwide lockdown period according to the COVID-19 pandemic in Korea during the early COVID-19 period was investigated, and the outpatient cancellation rates before and after the second vaccination were compared to determine the effect of COVID-19 vaccination.

### 2.4. Statistical Analysis

Logistic regression and Pearson’s chi-squared test were used for the statistical analysis. Group comparisons were also made in pairs using the chi-squared test, according to the Bonferroni corrections. Statistical significance was defined as a *p*-value of less than 0.05. All analyses were performed using SAS Enterprise Guide 6.1 (SAS Institute Inc., Cary, NC, USA).

## 3. Results

### 3.1. Patient Demographics

From 2019 to 2021, data from 121,042 patients who were scheduled to visit the Sanggye Paik Hospital Ophthalmology Department were obtained. Among these patients, 101,054 visited the outpatient clinic as scheduled, whereas 19,988 did not visit the scheduled outpatient clinic. The cancellation rate was 16.51% over the entire study period. By period, 35,380 patients visited the scheduled outpatient clinic during pre-COVID-19, whereas 6700 (15.92%) did not. During early COVID-19, 31,298 patients visited the clinic, whereas 7335 (18.99%) did not. A total of 34,376 patients came to the clinic during late COVID-19, whereas 5953 (14.76%) did not. Compared to that of the early COVID-19 period, the number of patients decreased, and the cancellation rate increased during the late COVID-19 period. In addition, the late COVID-19 period showed a lower cancellation rate than that of the pre-COVID-19 period (Table 1).

**Figure 1:**
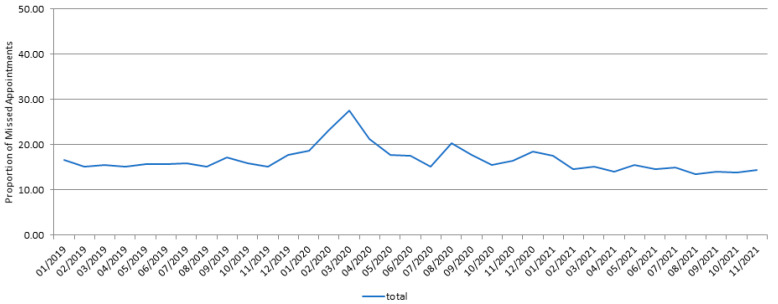
Changes in the cancellation rates among the overall ophthalmology outpatients.

Analyzed by month, the cancellation rate was higher in February and March 2020 of the early COVID-19 period compared to that of the other months, which coincided with the start of the COVID-19 pandemic in Korea. In addition, the cancellation rate increased in August and December 2020, which was the second pandemic outbreak in South Korea (Figure 1). The increase in the cancellation rate showed a decreasing trend with time, despite the COVID-19 pandemic. It can be inferred that patients became insensitive to the pandemic because of the prolonged coronavirus crisis.

### 3.2. Comparison of Each Department

**Figure 2:**
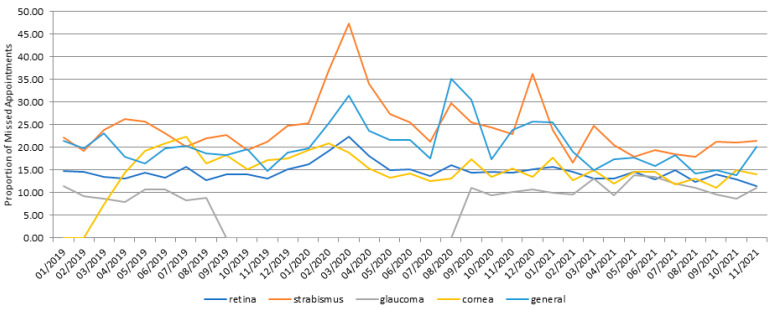
Monthly cancellation rates among different Ophthalmic Departments.

The departments were classified into general ophthalmology, cornea, glaucoma, retina, and strabismus/pediatric ophthalmology. General ophthalmology showed a cancellation rate of 20.15% over the entire period. In addition, the cancellation rate was 19.13% during pre-COVID-19, 24.13% during early COVID-19, and 17.34% during late COVID-19, showing a significant increase in the cancellation rate during the early COVID-19 period, and a significant decrease during the late COVID-19 period. The Retina Department showed a cancellation rate of 14.61% over three years. Divided by the periods, the cancellation rate was 14.08% during the pre-COVID-19 period, 16.03% during the early COVID-19 period, and 13.65% during the late COVID-19 period, displaying significant differences in all periods. The Cornea Department showed a cancellation rate of 15.40% over the entire period, with rates of 17.37%, 15.66%, and 13.68%, respectively, as described above. The cancellation tendency of this department was different from that of other departments, showing a decreasing cancellation rate during COVID-19. The Strabismus/Pediatric Ophthalmology Department showed the highest cancellation rate of 24.21% over the three years, with 22.68% during the pre-COVID-19 period, 29.82% during the early COVID-19 period, and 20.32% during the late COVID-19 period, with the highest cancellation rate during early COVID-19. The glaucoma division showed a cancellation rate of 10.66% for the total period, 10.11% for the pre-COVID-19 period, 10.56% for the early COVID-19 period, and 11.15% for the late COVID-19 period. Although its cancellation rate was lower and more constant than that of other departments, the statistical significance could not be retained, as the professor of the Glaucoma Department was absent from September 2019 to August 2020 due to overseas training (Figure 2).

### 3.3. Reasons for Cancellation and Patient Factors (Age, Gender, Cause, Insurance, and Residence)

**Figure 3:**
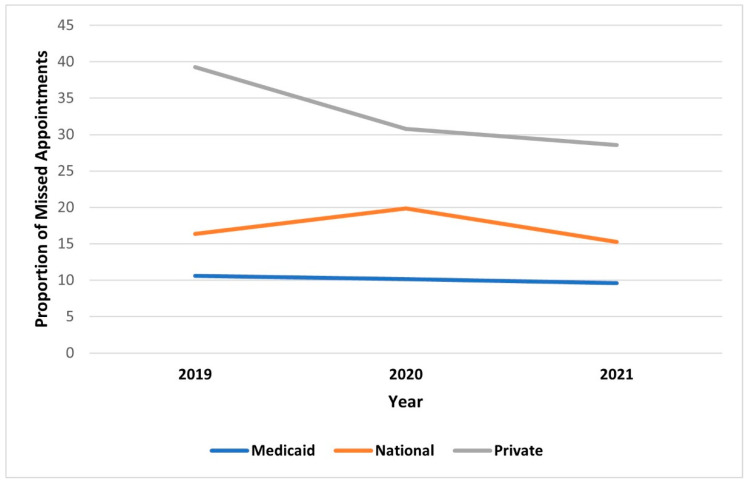
Cancellation rates among different insurance types.

Baseline characteristics, such as age, sex, and residence, of patients who did not visit the hospital on the scheduled date were analyzed. Patients between 60 and 80 years old accounted for the highest percentage of patients who did not visit the hospital during the entire study period. The proportion of patients under the age of 20 years who did not visit the hospital increased from 17.49% for pre-COVID-19 to 20.08% for early COVID-19. There were no differences in gender, but the ratio of cancelled patients who lived in the same administrative district as the hospital increased after COVID-19 (Table 2). Multiple regression analysis was conducted with factors such as gender, age, residence, type of insurance, and year. Consequently, gender and residence were excluded, as they did not show significant results. In comparison with differences in insurance types, the odds ratio of the cancellation rate of National Insurance was 1.680 compared to Medicaid. In the analysis according to year, the odds ratio of the cancellation rate in 2020 was 1.245 compared to 2019 (Table 3, Figure 3).

If the patient did not visit the hospital on the scheduled date, the hospital called the patient, and recorded the reason for not visiting the hospital. Analysis of the cause of not visiting the hospital showed that 42.91% of the cases failed to receive phone calls. There were no significant differences in the causes of hospital visits by period (Table 4 and Table 5).

## 4. Discussion

In this study, the COVID-19 pandemic increased the rate of missed clinic visits in the Ophthalmology Department during the early COVID-19 period (1 January 2020–31 December 2020). However, in the late COVID-19 period (1 January 2021–31 November 2021), the rate of no-show in the Ophthalmology Department recovered to that of the pre-COVID-19 period. The rise in no-show began about two weeks after the first COVID-19 case was confirmed on 20 January 2020. In March 2020, the no-show rate peaked at 27.6 3%, and subsequently improved two months through May 2020.

In Korea, previous studies have reported reduced patient visits due to the COVID-19 pandemic. Brant et al. described that missed visits to the Ophthalmology Department increased dramatically during the COVID-19 pandemic [4], and Aiello et al. reported that the rate of corneal transplantation was significantly reduced (−56%) compared with that in 2019 in Italy [9]. In India, during the COVID-19 lockdown, ophthalmologists were not seeing patients, and had near-total cessation of elective surgeries [7].

In our study, as in previous studies, the number of missed visits has increased since March 2020. In Korea, distancing protocols began on 6 May 2020, but the cancellation rate did not increase at that time. In addition, on 28 June 2020, the level of distancing protocols was raised, but the cancellation rate did not increase; on 11 November 2020, the level of distancing protocols was raised to the highest, but the cancellation rate did not increase significantly as well. Therefore, it can be concluded that the level of distancing protocols did not affect the cancellation rate in Korea. Nair et al. reported that during the lockdown period from 24 March 2020, all regular outpatient consultations except those for emergency patients were discontinued in India [7]. The assumed reason why the distancing protocol in Korea did not affect the cancellation rate is because it was not applied to the public transportation system. Therefore, there was no problem with the transport of the patients, and those visiting our hospital mainly lived in the same administrative district as the hospital. In addition, there were no restrictions on movement between administrative districts in Korea’s distancing protocols. Evidently, despite the COVID-19 pandemic, the increment of the cancellation rate decreased with time, which can be thought to be patients being somewhat insensitive to the pandemic due to the prolonged COVID-19 crisis.

The cancellation rate increased in all departments, but it was dramatically higher in the Department of General Ophthalmology and Strabismus/Pediatric Ophthalmology compared to that of the other departments. Other studies have reported that the no-show rates of the Department of Strabismus/Pediatric Ophthalmology, as well as the Department of Cornea, are high [4]. The difference in this study was that the cancellation rate of the Cornea Department decreased in the early COVID-19 period compared to that of the pre-COVID-19 period. The reason for this result is that the patients who had made reservations for surgery 5–6 months before, and those under postoperative examinations, account for a large proportion of the Cornea Department visits. If the patient failed to visit the hospital on the reservation date, the surgery would be booked again a few months later. Therefore, these patients are more likely to visit the hospital despite the risk of COVID-19. The cancellation rate in the Department of Retina or Glaucoma may be lower because the diseases treated in these departments are more likely to progress to irreversible vision loss than in the Department of General Ophthalmology or Strabismus/Pediatric Ophthalmology [4]. The pediatric ophthalmology has a relatively low disease severity due to the large number of children who regularly examine their visual acuity and strabismus. It is thought that the cancellation rate was higher because these children had to be followed by their parents on examinations.

Asian no-show rates were reported to be high in previous studies, with more risk-averse behavior and the fear of encountering racism pointed as possible reasons [4]. However, the early COVID-19 cancellation rate in this study, which only included Asians, was 18.99%, lower than the reported rate of 51.3% in a previous study. Although the cultural differences and the level of the distancing protocols should be considered, we believe that the higher no-show rate of Asians identified in previous studies is more likely caused by the fear of encountering racism.

Those between 60 and 80 years old accounted for the largest proportion of patients who did not visit the hospital over the entire period. In addition, the proportion of patients younger than 20 years who did not visit the hospital increased from 17.49% in the pre-COVID-19 period to 20.08% in the early COVID-19 period. This is consistent with the high cancellation rate of strabismus/pediatric ophthalmology during COVID-19. In addition, the reason for no significant increase in patients between 60 and 80 years old in the pre-COVID-19 and early COVID-19 periods was the effect of age-related vision-threatening diseases, such as age-related macular degeneration and glaucoma. There were no differences in sex. In addition, the proportion of patients living in the same administrative area as the hospital increased after COVID-19. The area of the administrative district (Nowon-gu) where the hospital is located measures approximately 35.44 km^2^, and the proportion of patients in the same administrative district among the no-show patients was 40.88%, which increased to 43.03% in early COVID-19, and decreased again to 41.17% in late COVID-19.

Public transportation, such as subways and public buses, is convenient to use in the same administrative district, and accessibility is, therefore, good. However, it is relatively inconvenient to access the hospital outside the administrative district. Given that there were no restrictions on public transportation in the distancing protocols of Korea, it seems that the differences were not significant among the various residential areas. Patients with relatively less severe diseases who visit the Department of General Ophthalmology or Strabismus/Pediatric Ophthalmology tend to visit hospitals close to their homes, which may cause these phenomena. The National Health Insurance system is a public medical insurance system in South Korea that is obligatory for most citizens. The low cancellation rate of Medicaid patients may be due to the low cost burden to these patients when visiting the hospital. In the case of patients with private insurance without national medical insurance, the cancellation rate was higher because the cost burden of visiting the hospital was higher than that of patients with Medicaid and National Insurance. The results suggest that when visiting the hospital was difficult due to the COVID-19 pandemic, the higher cost burden of visiting hospitals led to the higher cancellation rate. In South Korea, COVID-19 vaccinations for the general population began sequentially in April 2021 for those aged 75 or older. The vaccination status could not be confirmed because the data were collected retrospectively. However, it can be inferred from the steady decrease of the cancellation rate since April 2021 that the vaccination had significant effects. There were no significant differences in gender, age, insurance, and residential area between the groups that visited and did not visit the hospital. It can be inferred that the COVID-19 pandemic mostly affected the cancellation rate rather than other socioeconomic factors. This study has several limitations. First, this study cannot represent the entire population of Korea because it was a single-center study. Second, due to the hospital’s personal information protection policy, the classification of patients’ residences was inevitably classified as the ‘same administrative district’ or ‘different administrative district’. Therefore, analysis based on the distance from the residence could not be performed. Third, the Department of Ophthalmic Plastic/Reconstructive Surgery could not be analyzed because the department was absent in our hospital. Fourth, the statistics of the Department of Glaucoma were not significant because the sole professor of this department had overseas training from September 2019 to August 2020. Fifth, because we used questionnaires on reasons for not attending the clinic, the analysis might have been affected by recall bias. Sixth, we did not attempt to provide teleconsultations or over-the-phone consultations. Finally, the effect of vaccination on the cancellation rate could not be analyzed.

## 5. Conclusions

The COVID-19 pandemic has caused an overall decrease in ophthalmic outpatients. The Departments of General Ophthalmology and Strabismus/Pediatric Ophthalmology were the most affected by the pandemic. However, after about a year, the number of outpatients in these departments recovered to the level before the COVID-19 outbreak.

## Figures and Tables

**Table 1:** Changes in the cancellation rates among the overall ophthalmology outpatients.

Year	Cancellation	Visit	Cancellation Rate	*p*-Value
2019	6700	35,380	15.92%	<0.001 *
2020	7335	31,298	18.99%	
				<0.001 ^†^
2021	5953	34,376	14.76%	

* Chi-squared test between 2019 and 2020. ^†^ Chi-squared test between 2020 and 2021.

**Table 2:** Differences in age, gender, residence, and other patient factors by period (pre-COVID-19, early COVID-19, and late COVID-19).

	Pre-COVID-19	Early COVID-19	Late COVID-19	*p*-Value (Pre vs. Early) *	*p*-Value (Early vs. Late) ^†^
Sex				0.307	0.208
Male	49.66%	48.79%	49.89%		
Female	50.34%	51.21%	50.11%		
Age				<0.001	0.012
<20	17.49%	20.08%	17.79%		
20–39	7.09%	6.03%	6.69%		
40–59	26.04%	23.14%	23.72%		
60–79	39.07%	40.60%	41.14%		
>80	10.30%	10.16%	10.67%		
Residence				0.01	0.031
Nowon-gu	40.88%	43.03%	41.17%		
Others	59.12%	56.97%	58.83%		
Insurance type				0.143	0.005
Public	94.03%	94.79%	93.46%		
Medicaid	5.64%	4.94%	6.20%		
Private	0.33%	0.27%	0.34%		

* Chi-squared test between 2019 and 2020. ^†^ Chi-squared test between 2020 and 2021. COVID-19, coronavirus disease.

**Table 3:** Logistic regression of sociodemographic data for cancellation rate.

	Odds Ratio	*p*-Value	Confidence Interval	
			Lower	Upper
Age	0.990	<0.001	0.990	0.991
Insurance type				
Medicaid				
National	1.680	<0.001	1.575	1.792
Private	3.513	<0.001	2.573	4.796
Year				
2019				
2020	1.245	<0.001	1.200	1.291
2021	0.915	<0.001	0.881	0.951

**Table 4:** Reasons for not visiting the clinic.

	Examinations in Higher Grade Hospital	No Improvement of Symptoms
		Rejudgment in Another Hospital
Hospital factors	Complaints about medical staff (lack of explanation, unfriendliness)	
	Dissatisfaction with administrative procedures (medical record copy, CD copy, medical certificate, inconvenience in receiving)	
	Complaints about facilities of the hospital (elevators and parking)	
	Delays in examination and treatment	
	Demands for expert ophthalmic clinic	
	Delays in medical image interpretations	
	Reservation change to same-day reception	
Patient factors	Personal reasons (family events, business trip, and overseas trip)	
	Spare medications	
	Lack of proper appointment time	
	Simple cancellations	
	Improvement of symptoms	
	Lack of examinations	
	Overlapping appointments	
	Moving to other regions	
	Cost burden	
	Forgetting the appointment	
Contact factors	Errors of contact numbers	
	Failure to receive phone calls	
Hospitalization in other departments		
Death		
Hospitalization in other hospitals		

**Table 5:** Distribution of cancellation reasons by year.

	Pre-COVID-19	Early COVID-19	Late COVID-19	*p*-Value (Pre vs. Early) *	*p*-Value (Early vs. Late) ^†^
Hospital factors	6.01%	6.04%	6.11%	0.032	<0.181
Patient factors	49.09%	49.60%	48.83%		
Contact factors	42.76%	42.89%	43.10%		
Others	2.13%	1.47%	1.84%		

* Chi-squared test between 2019 and 2020. ^†^ Chi-squared test between 2020 and 2021. COVID-19, coronavirus disease.

## Data Availability

All data generated or analyzed during this study are included in this article. Further enquiries can be directed to the corresponding author.
